# A Study on the Relationship Between Physical Activity, Sedentary Behavior, and Sleep Duration in Preschool Children

**DOI:** 10.3389/fpubh.2021.618962

**Published:** 2021-04-07

**Authors:** Zhenya Chang, Wei Lei

**Affiliations:** ^1^College of Physical Education and Health, East China Normal University, Shanghai, China; ^2^Preschool Education School, Changsha Normal University, Hunan, China; ^3^College of Physical Education, Jiangxi Science and Technology Normal University, Jiangxi, China

**Keywords:** movement behavior, accelerometer, child, health, sleep

## Abstract

**Purpose:** Explore the role of physical activity (PA) and sedentary behavior (SB) in the sleep of preschool children.

**Method:** Preschool children (*n* = 458) from kindergarten were recruited, measures included accelerometer-derived 24 h activity [SB, light physical activity (LPA), moderate-to-vigorous physical activity (MVPA), daytime sleep duration (DSP), and nocturnal sleep duration (NSP)]. A self-made questionnaire was used to supplementary survey on different types of SB. The two-layer chi-square test and the Cochran–Mantel–Haenszel test were used and significance set at *p* < 0.05.

**Results:** PA is mainly a protective factor for DSP, while SB is mainly a risk factor for NSP of preschool children; Screen-type SB including screen viewing SB and video game SB is a risk factor for NSP, while quiet learning SB have no significant correlation with sleep duration in preschool children; Gender and social economic status are important factors affecting the association of PA, SB, and sleep duration in preschool children.

**Conclusions:** The relationship between sleep duration at different periods and PA and SB of preschool children is different. Future educators need to gradually realize the different factors affecting the sleep of preschool children at different stages, clarify the protective factors and risk factors, in order to improve the sleep duration of preschool children.

## Introduction

Sleep plays an important role in early childhood growth and health, Preschool children with insufficient sleep time are more prone to obesity or growth disorders and have a higher risk of psychological problems (1). A Joint Consensus Statement of the American Academy of Sleep Medicine and the American Sleep Medicine Foundation that preschool children (3–5 years old) need 10–13 h of sleep every day. However, relevant studies have shown that the sleep duration of preschool children in China is not optimistic. The results of the Shanghai 10,000 People Questionnaire report show that the sleep duration of preschool children in Shanghai is short, with an average sleep time of 9.06 h, which is less than the average sleep time of 9.57 h in 10 other cities of the country (2). Therefore, it is of great significance to pay attention to the sleep duration of preschool children to promote children's sleep quality.

There are many factors that affect the sleep duration, such as parenting practices and expectations, family habits, cultural preferences, and day care schedules. With the increasing convenience of social life, people's sedentary behavior (SB) is becoming more and more serious, and SB has gradually become one of the most important factors to promote the improvement of sleep quality. Related research shows that school-age children with adequate physical activity (PA) are more likely to get adequate sleep than who with lower PA (3–5). However, some scholars found that the increase in moderate to vigorous physical activity (MVPA) in children and the decrease in SB are not related to sleep duration or even the inverse relationship (6). The above contradictory research results are largely due to the subjectivity of the assessment tools. Although sleep and physical activity can be evaluated through questionnaire, but the accuracy may be greatly affected and this problem is more complicated in preschool children who need to be evaluated by parents (7). In recent years, accelerometers and other object evaluation tools have been increasingly used for the evaluation of PA and SB. Even now people gradually believe that accelerometers are the “gold standard” for PA evaluation. Later, people found that monitoring time was an important factor affecting the evaluation effect and in addition to the promulgation of 24-H Movement Guidelines recently, researchers began to emphasize the need for accelerometers to be worn for 24 h, thus creating an opportunity for sleep and PA to be evaluated with the same tool instead of being evaluated separately as in the past.

Scholars have followed this development trend and used a 24-h monitoring model to explore the association between PA, SB, and sleep duration in preschool children. Some of these scholars found nocturnal sleep duration (NSP) was related to SB or light physical activity (LPA), but not to MVPA (8), while others found NSP is related to SB, but not to PA (9). Recently, some scholars believed that NSP not only related to PA but also SB (10). The above research systematically revealed the association between NSP, PA, and SB of preschool children. However, the results were inconsistent, and at least two problems remained to be resolved. First, daytime sleep duration (DSP) is the necessary components of the total sleep duration (TSP), how does it correlate with PA or SB. Second, how do different types of SB relate to the sleep duration of preschool children. Different types of SB may have different effects on sleep duration. Under this background, this study is to further explore the comprehensive relationship between DSP, NSP, TSP, PA, and SB of preschool children in order to enrich and supplement the relevant evidence of 24-h movement guidelines.

## Materials and Methods

### Participants

Following ethical clearance from the corresponding authors institutional review board (HR006-2019). Firstly, based on economic development levels and the principle of stratified sampling, samples were selected from nine kindergartens. Secondly, 10 children (five boys and five girls) are planned to recruit in each particular age bracket (3, 4, 4.5, 5, 5.5, and 6) in each kindergarten. In actual recruitment process, appropriate adjustments in the number of children of different age groups can be made according to the actual situation of kindergartens. Finally, 530 children participated in this research. According to the relevant data filtering criteria, test data of 72 children's movement behavior were incomplete or invalid. The final group, therefore, consisted of 458 children (Table 1).

**Table 1:** List of basic conditions of the participants.

		**Sex**		**Sum**
		**Male**	**Female**	
Age	3	22	20	42
	3.5	28	38	66
	4	35	41	76
	4.5	44	41	85
	5	41	44	85
	5.5	35	37	72
	6	21	11	32
Sum		226	232	458

### Physical Activity, Sedentary Behavior, and Sleep Duration

A three-axis accelerometer (ActiGraph GT3X-BT) (Pensacola, FL, USA) was used to evaluate the movement behavior. Preschool children should wear the accelerometer on the right iliac crest and carry it for 7 consecutive days a week (5 weekdays and 2 weekend days). Parameter setting details of the accelerometer can be seen in Table 2. After testing, Data were downloaded and initially analyzed by using ActiLifev6.13.4. The mode of low frequency extension was enabled to improve the sensitivity of monitoring while dealing with ActiGraph sleep data (11). Visual diagnosis by experts per minute was used to assess preschool children's daytime sleep duration (12, 13).

**Table 2:** Measurement parameter settings list of GT3X-BT.

**No**.	**Parameter content**	**Setting**	
1	Test instrument	ActiGraph GT3X-BT	
2	Sampling interval^a^	15s	
3	Definition of not wearing	Choi algorithm (2011)	
4	Minimum time of wearing per day	≧1,000 min	
6	Minimum days of wearing	At least 4 days, including 3 weekdays and 1 weekend	
7	Different values of	SB	0–467
	intensity (Counts)^a^	LPA	468–2,207
		MPA	2,208–3,991
		VPA	3,992及以上
8	Sleep time algorithm	Tudor-Locke algorithm (2014)	

*SB means sedentary behavior; LPA means light physical activity; MPA means moderate physical activity; VPA means vigorous physical activity*;

a *(26)*.

### Different Types of SB

Drawing on the European ToyBox project (http://www.toybox-study.eu) survey method of SB types, SB is divided into three parts: quiet learning SB, screen viewing SB, and video game SB.The sum of screen viewing SB and video game SB is equal to screen time which in the 24 h movement guidelines for preschoolers. The specific survey questions are: About how many hours a day does your child have quiet play (looking into books, playing with blocks, playing with dolls, drawing, and construction) during leisure time? About how many hours a day does your child usually watch television (including DVDs and videos) in his/her free time? About how many hours a day does your child use the computer for activities like playing games on a computer, game consoles (e.g., Playstation, Xbox, and GameCube) during leisure time? According to the above questions, parents ticked out the options for SB types during the week and the weekend, respectively. The option setting method is based on the time setting method and calculation method of the Early Years Physical Activity Questionnaire (14). It is set to 10 items [(1) No; (2) <15 min per day; (3) 16–30 min per day; (4) 31 min to 1 h per day; (5) 1–2 h per day; (6) 3 h per day to 4 h; (7) 5–6 h per day; (8) 7–8 h per day; (9) 8 h or more per day; (10) I don't know]. After checking, the parents will estimate the daily duration of SB according to the maximum duration of the options, such as 15, 30, 60 min, etc., because parents tend to over-report PA levels and under-report SB (15).

### Social Economic Status

Refer to the existing research (16) and compile a questionnaire on the Social Economic Status (SES). It included parental education level, parental occupation and household income. Then according to the method of calculating SES in International Students Evaluation Projects, SES was calculated in four steps. For the category marked “Occupation,” it was carried out by the scoring criteria from the occupational classification index of international SES by Ganzeboom and Treiman (17). At last, SES was eventually classified into three grades, namely, low, medium, and high SES according to the method of standard deviation (18).

### Statistical Analysis

Use IBM SPSS Statistics 25.0 software for statistical processing. In order to determine the normality of continuous variables, the histogram, skewness, and kurtosis are combined for comprehensive consideration. According to SES and gender, the individual measurement characteristics of the subjects and the statistical information of important variables in the study are presented in turn. The stratified chi-square test and the Cochran–Mantel–Haenszel test were used to explore the protective factors and risk factors of sleep duration of children of different genders and different SES.

## Results

### The Basic Situation of the Study

According to the recommended standard of “Guidelines on PA, SB, and sleep for children under 5 Years of age” issued by the World Health Organization on May 10, 2019, the compliance rate of TSP of preschool children was 66.6%, of which boys' compliance rate was 64.2%, girls' compliance rate was 69.0%, low SES children' compliance rate was 62.1%, medium SES children' compliance rate was 67.1%, High SES children' compliance rate was 69.1%. The preschool children's MVPA compliance rate was 41.7%, TPA compliance rate was 92.6%, of which boys and girls MVPA compliance rate was 49.1 and 34.5%, TPA compliance rate was 95.6 and 89.7%. The MVPA compliance rate of children with low, middle and high SES was 47.0, 40.1, and 45.5%, and the TPA compliance rate was 92.4, 92.6, and 92.7%. Individual characteristics and important variables are presented in the form of mean (standard deviation), as shown in Table 3. In addition, after calculation, SES = (0.646^*^Z occupation + 0.636^*^Z education level + 0.765^*^Z household income)/2.104, and then the SES level is divided into three levels according to the method of 1 standard deviation up and down (17), therefore, the proportion of preschool children from low, medium, and high SES families in this study was 14.4, 73.6, and 12%, respectively.

**Table 3:** Basic situation of important variables in preschool children.

		**SES**				**Sex**			**Total**
		**Low**	**Medium**	**High**	***F***	**Male**	**Female**	***t***	
Height (cm)		107.9 (7.1)	107.8 (6.9)	106.3 (8.2)	1.04	108.2 (7.0)	107.1 (7.1)	2.83	107.6 (7.1)
Weight (kg)		17.8 (3.0)	17.9 (3.1)	17.8 (3.5)	0.03	18.2 (3.4)	17.7 (2.9)	2.84	17.9 (3.1)
BMI		15.3 (1.4)	15.4 (1.6)	15.7 (1.6)	1.05	15.4 (1.7)	15.4 (1.5)	0.12	15.4 (1.6)
SP	DSP (min)	**65.5 (26.1)**	71.5 (23.4)	**77.9 (24.7)**	4.02^*^	70.0 (26.4)	73.0 (21.7)	1.63	71.4 (24.1)
	NSP (min)	547.1 (34.8)	546.0 (36.7)	540.0 (34.0)	0.73	544.8 (35.7)	546.1 (36.5)	0.14	545.4 (36.1)
	TSP (min)	612.6 (37.7)	617.5 (37.0)	617.9 (32.3)	0.52	614.7 (35.5)	618.9 (37.3)	1.49	616.8 (36.4)
SB (min)		495.6 (58.5)	486.6 (47.3)	478.2 (55.8)	1.82	**480.0 (50.1)**	**493.6 (49.5)**	8.56^**^	486.9 (50.2)
PA	LPA (min)	179.2 (31.4)	184.7 (28.6)	186.5 (31.3)	1.18	**189.7 (29.8)**	**178.7 (27.9)**	16.63^***^	184.1 (29.4)
	MVPA (min)	56.7 (22.0)	56.0 (17.7)	59.5 (19.7)	0.87	**60.8 (19.2)**	**52.3 (17.0)**	24.95^***^	56.5 (18.6)
	TPA (min)	235.9 (47.0)	240.7 (39.7)	246.0 (41.4)	0.91	**250.5 (41.8)**	**231.0 (38.7)**	26.80^***^	240.6 (41.4)
Length of time not worn (min)		96.0 (38.2)	95.2 (42.0)	97.8 (43.5)	0.10	94.8 (39.3)	96.4 (43.8)	0.19	95.7 (41.6)

*SB means sedentary behavior; LPA means light physical activity; MVPA means moderate-to-vigorous Physical Activity; TPA means total of physical activity; Bold indicates that there is a significant difference between the two*,

* *represents p < 0.05*,

** represents p < 0.01, and

*** *represents p < 0.001*.

### Correlation Between PA, SB, and Sleep Duration of Children of Different Genders

For boys, LPA and MVPA are protective factors for DSP, however, NSP and TSP are not affected by the level of PA and SB. For girls, SB is a risk factor for NSP, TPA is a protective factor for NSP, and LPA, MVPA, and TPA are protective factors for TSP. For the overall preschool children, SB is a risk factor for NSP, LPA is a protective factor for TSP, MVPA is a protective factor for TSP and DSP, and TPA is a protective factor for TSP (see Table 4).

**Table 4:** Protective and risk factors of adequate sleep for children of different genders.

**Type of sleep duration**	**Variables**	**Sex**	**OR (95%CI)**
Total sleep time (≧600 min/day)	SB (≦25th percentile)	Male	1.679 (0.895,3.148)
		Female	1.203 (0.602,2.402)
		Total	1.414(0.889,2.249)
	LPA (≧75th percentile)	Male	0.563 (0.316,1.003)
		Female	0.408 (0.208,0.799)^**^
		Total	0.481 (0.312,0.743)^**^
	MVPA (≧60 min/day)	Male	0.722 (0.419,1.247)
		Female	0.492 (0.277,0.874)^*^
		Total	0.589 (0.398,0.872)^**^
	TPA (≧180 min/day)	Male	0.758 (0.191,3.016)
		Female	0.179 (0.041,0.784)^*^
		Total	0.322 (0.122,0.848)^*^
Nighttime sleep duration (≧75th percentile)	SB (≦25th percentile)	Male	1.018 (0.535,1.940)
		Female	3.010 (1.525,5.941)^**^
		Total	1.705 (1.072,2.711)^*^
	LPA (≧75th percentile)	Male	0.689 (0.358,1.327)
		Female	0.584 (0.244,1.399)
		Total	0.682 (0.407,1.142)
	MVPA (≧60 min/day)	Male	0.879 (0.489,1.578)
		Female	0.777 (0.401,1.505)
		Total	0.867 (0.564,1.335)
	TPA (≧180 min/day)	Male	0.877 (0.219,3.504)
		Female	0.365 (0.152,0.878)^*^
		Total	0.512 (0.247,1.058)
Daytime sleep duration (≧75th percentile)	SB (≦25th percentile)	Male	1.497 (0.802,2.795)
		Female	1.474 (0.722,3.008)
		Total	1.523 (0.954,2.430)
	LPA (≧75th percentile)	Male	0.415 (0.205,0.840)^*^
		Female	0.867 (0.387,1.945)
		Total	0.592 (0.349,1.005)
	MVPA (≧60 min/day)	Male	0.446 (0.244,0.815)^**^
		Female	0.562 (0.280,1.128)
		Total	0.524 (0.334,0.823)^**^
	TPA (≧180 min/day)	Male	0.564 (0.154,2.069)
		Female	0.537 (0.216,1.335)
		Total	0.588 (0.281,1.230)

*SB means sedentary behavior; LPA means light physical activity; MVPA means moderate-to-vigorous Physical Activity; TPA means total of physical activity*.

* *Represents p < 0.05*,

** represents p < 0.01, and

*** *represents p < 0.001*.

### The Correlation of PA, SB, and Sleep Duration of Children With Different SES

SB is a risk factor for DSP in children with low SES and a risk factor for NSP in children with medium SES; LPA is a protective factor for DSP and TSP for children with medium SES, MVPA is a protective factor for DSP for children with medium SES; PA and SB were not significantly associated with sleep duration in children with high SES (see Table 5).

**Table 5:** Protective and risk factors of adequate sleep for children of different social economic status.

**Type of sleep duration**	**Variables**	**SES**	**OR (95%CI)**
Total sleep time (≧600 min/day)	SB (≦25th percentile)	Low	2.172 (0.614,7.684)
		Medium	1.295 (0.730,2.298)
		High	2.427 (0.589,9.996)
	LPA (≧75th percentile)	Low	0.364 (0.109,1.218)
		Medium	0.439 (0.258,0.748)^**^
		High	1.218 (0.361,4.313)
	MVPA (≧60 min/day)	Low	0.722 (0.266,1.960)
		Medium	0.642 (0.369,1.040)
		High	0.646 (0.205,2.041)
	TPA (≧180 min/day)	Low	0.385 (0.041,3.659)
		Medium	0.306 (0.088,1.060)^*^
		High	1.118 (1.002,1.246)
Nighttime sleep duration (≧75th percentile)	SB (≦25th percentile)	Low	1.294 (0.378,4.433)
		Medium	2.172 (1.237,3.815)^**^
		High	1.055 (0.236,4.720)
	LPA (≧75th percentile)	Low	0.673 (0.164,2.757)
		Medium	0.659 (0.359,6.070)
		High	1.476 (0.407,1.142)
	MVPA (≧60 min/day)	Low	1.182 (0.400,3.496)
		Medium	0.798 (0.471,1.352)
		High	1.250 (0.317,4.929)
	TPA (≧180 min/day)	Low	0.217 (0.033,1.427)
		Medium	0.581 (0.234,1.441)
		High	0.911 (0.832,0.998)
Daytime sleep duration (≧75th percentile)	SB (≦25th percentile)	Low	3.543 (1.043,12.040)^*^
		Medium	1.173 (0.641,2.148)
		High	1.200 (0.357,4.030)
	LPA (≧75th percentile)	Low	1.333 (0.354,5.026)
		Medium	0.462 (0.229,0.932)^*^
		High	0.632 (0.185,2.155)
	MVPA (≧60 min/day)	Low	1.174 (0.380,3.622)
		Medium	0.476 (0.268,0.847)^*^
		High	0.413 (0.128,1.328)
	TPA (≧180 min/day)	Low	1.304 (0.135,12.593)
		Medium	0.507 (0.204,1.262)
		High	0.500 (0.065,3.863)

*SB means sedentary behavior; LPA means light physical activity; MVPA means moderate-to-vigorous Physical Activity; TPA means total of physical activity*.

* *Represents p < 0.05*,

** represents p < 0.01, and

*** *represents p < 0.001*.

### The Relationship Between Different Types of SB and the Sleep Duration of Preschool Children

Screen-type SB is a risk factor for NSP, and there is no significant correlation with the sleep duration at other levels; Quiet learning SB was not significantly associated with the sleep duration of preschool children at all levels. Since the Test of Homogeneity of Odds Ratio results suggest that the OR values between different genders and different SES layers are homogeneous (*p* > 0.05), the relevant stratified data is no longer presented separately (see Table 6).

**Table 6:** Protective and risk factors of adequate sleep for children of different sedentary behavior.

**Type of sleep duration**	**SB type**	**OR (95%CI)**
Total sleep time (≧600 min/day)	Screen type SB (≦60 min/day)	1.022 (0.653,1.601)
	Quiet Learning SB (≦25th percentile)	0.967 (0.655,1.427)
Nighttime sleep duration (≧75th percentile)	Screen type SB (≦60 min/day)	1.803 (1.135,2.863)^*^
	Quiet Learning SB (≦25th percentile)	0.978 (0.640,1.493)
Daytime sleep duration (≧75th percentile)	Screen type SB (≦60 min/day)	1.281 (0.797,2.058)
	Quiet Learning SB (≦25th percentile)	0.849 (0.555,1.300)

*SB means sedentary behavior; LPA means light physical activity; MVPA means moderate-to-vigorous Physical Activity; TPA means total of physical activity; the screen type SB refers to the sum of the screen viewing type SB and the video game type SB, and is the same as the screen time in the guide*.

* *Represents p < 0.05*,

** represents p < 0.01, and

*** *represents p < 0.001*.

## Discussion

This study used Actigraph triaxial accelerometers to monitor PA, SB, and sleep duration of preschool children in China for 24 h. The results found that PA is more closely related to DSP and is a protective factor for DSP, while SB is more closely related to NSP and is a risk factor for NSP; Screen-type SB are risk factors for NSP in preschool children, and quiet learning SB have no significant correlation with sleep duration at all levels in preschool children; Gender and SES are important influencing factors for preschool children's movement behavior.

### Correlation Between PA, SB, and NSP

The results of this study are consistent with the results of previous studies (9). NSP has nothing to do with PA, it is related to SB which is a risk factor for preschool children' NSP. This study further reveals screen time, Including screen viewing SB and video game SB are also risk factors for NSP. The screen time of preschool children, especially the screen time before going to bed, may have an adverse effect on sleep duration. For every hour of screen time increase, the NSP will be shortened by 3 min (95%CI: 0.6–5), and the sleep latency period is extended by 1.6 min (95%CI: 0.59–2.63) (19). However, this study is different from previous studies that used preschool children in the United States as research subjects (9). It was found that there was a gender difference in the association between SB and NSP of preschool children in China. There was a significant association in girls but not in boys. This may be due to the differences in the characteristics of SB between 3 and 6 years old boys in China and the United States which in turn leads to different associations between SB and sleep duration. Of course, it may also be due to the different cutoff values for SB used. Existing studies have shown that different cutoff values will have a significant impact on the data collected by the accelerometer (20). In this study, it may be more accurate to use the cut-off value of SB established for preschool children in China to evaluate the characteristics of SB in preschool children in China. Some scholars explored the relationship between SB and sleep duration of preschool children in Hong Kong, China (10). However, the study did not provide information about the gender differences, but what can be determined is the study is consistent with the study by Williams et al. (8), found a positive correlation between PA and NSP, the latter also found in the study that the role of PA is LPA, not MVPA. This study found that TPA is a protective factor for NSP in girls. However, after splitting TPA into LPA and MVPA, no significant correlation was found between the two and NSP. The PA of preschool children is characterized by LPA, mixed with MVPA. Therefore, LPA and MVPA may have a certain correlation with NSP, resulting in a significant correlation between TPA and NSP. As for why only TPA for girls has a significant association with NSP, it may be that the PA level of girls is significantly lower than that of boys, which in turn leads to more obvious health benefits of PA for sleep. In addition, this study further reveals that the association between PA, SB, and sleep duration will be affected by gender and SES. Future educators need to formulate corresponding education strategies based on this feature.

### Correlation Between PA, SB, and Daytime Sleep Duration

Unlike the association mechanism of NSP, physical activity is closely related to DSP and is significant in boys, while SB is not significantly related to DSP (see Table 4). It can be seen that PA is relatively closely related to DSP, while SB is relatively closely related to NSP. Preschool children are tired after experiencing high levels of PA during the day or the neural mechanism changes from very excited to quiet, which is conducive to the increase in DSP, and the close relationship between NSP and SB may be due to the large amount of screen time, the inverse association between screen time and sleep duration has been confirmed by many studies (21, 22). From the perspective of SES, SB is a risk factor for children with low SES, and LPA and MVPA are protective factors for children with medium SES. It is consistent with the existing research which show that compared with children with high SES, children with low SES exhibit lower PA levels and longer SB (23). The results of this study also show that SB decreases with the improvement of SES (see Table 3). Future scholars need to pay attention to families with low SES of sitting too long or lack of PA.

### Correlation Between PA, SB, and Total Sleep Duration

Perhaps the correlation between PA, SB, and TSP of preschool children is more meaningful for practical guidance. After all, DSP is closely related to the NSP. DSP is an important supplement to insufficient NSP. According to the results of this study, DSP was significantly negatively correlated with the NSP (*r* = −0.320, *p* < 0.001). Scholar Wen (21) explored the relationship between PA and sleep in preschool children in Shanghai, China based on a triaxial accelerometer and a sleep questionnaire, only to find that MPA and MVPA in girls were significantly associated with insufficient sleep, and that physical activity in boys was not significantly associated with insufficient sleep. LPA is also not significantly associated with the sleep duration in preschool children. It is worth noting that the sleep duration assessment in this study was conducted using only one option in the Achenbach Child Behavior Checklist (1.5–5, CBCL) “sleep less than most children,” without distinguishing between DSP and NSP. This study made full use of three-axis accelerometer to comprehensively monitor PA, SB, and sleep duration of preschool children, and further divided sleep duration into DSP and NSP. The results not only found a significant association between MVPA and TSP, but also found a significant association between LPA and TSP of preschool children. Both LPA and MVPA are protective factors of TSP of preschool children. Similar to Wen's research, the significance of this association was found only in girls. As a result, future educators should gradually realize the impact of gender differences on the correlation between PA and sleep duration, and gradually realize the health benefits of LPA, not just emphasize the health benefits of MVPA.

Related research shows that SES is associated with PA and sleep of preschool children, especially low SES families (24). In addition, there is a similar finding in the adolescent group, there is an inverse correlation between SES and adolescent sleep quality, children with low SES have poor sleep quality and short duration (25). The results of this study are consistent with previous studies, and TSP of preschool children with high, medium, and low SES sequentially decreased (see Table 3). In addition, this study also found that PA is a protective factor for the TSP of children with medium SES. PA does not have a significant role in regulating the total sleep duration of children with low SES and high SES. Future scholars need to further explore low SES and high SES factors affecting children's sleep duration.

In addition, the average total sleep time of preschool children was about 10.3 h, which was higher than the research results of the Shanghai 10,000 People Questionnaire Survey and met the recommended amount of relevant guidelines (10–13 h). However, the satisfaction rate of reaching the recommended amount is only 66.6%, which means that about 33.4% of preschool children have a total sleep time of <10 h, so it is urgent to improve the sleep time of related preschool children.

## Limitations

First, although the accelerometer can be used to measure PA and sleep at the same time, the use of the same equipment to evaluate PA, SB, and sleep in preschool children is still scientifically limited, and the accelerometer is still more suitable for measuring PA, future scholars can use more accurate and convenient equipment to measure sleep. Second, this study is a cross-sectional study, and it is difficult to fully reflect the accurate correlation between PA, SB, and sleep. Future scholars need to further investigate the longitudinal correlation between PA, SB, and sleep duration in longitudinal studies.

## Conclusions

The relationship between sleep at different periods and PA and SB of preschool children is different. Future educators need to gradually realize the different factors affecting the sleep of preschool children at different times, clarify the relevant protective factors and risk factors, in order to better improve the sleep duration of preschool children.

## Data Availability Statement

The original contributions presented in the study are included in the article/supplementary materials, further inquiries can be directed to the corresponding author/s.

## Ethics Statement

The studies involving human participants were reviewed and approved by Human Subjects Protection Committee of East China Normal University. Written informed consent to participate in this study was provided by the participants' legal guardian/next of kin.

## Author Contributions

ZC is responsible for the design, research, and writing of the article. WL is responsible for the language editing and external support of the article. All authors contributed to the article and approved the submitted version.

## Conflict of Interest

The authors declare that the research was conducted in the absence of any commercial or financial relationships that could be construed as a potential conflict of interest.
